# Agrowaste-generated biochar for the sustainable remediation of refractory pollutants

**DOI:** 10.3389/fchem.2023.1266556

**Published:** 2023-11-16

**Authors:** Sougata Ghosh, Maitri Nandasana, Thomas J. Webster, Sirikanjana Thongmee

**Affiliations:** ^1^ Department of Physics, Faculty of Science, Kasetsart University, Bangkok, Thailand; ^2^ Department of Microbiology, School of Science, RK University, Rajkot, Gujarat, India; ^3^ School of Health Sciences and Biomedical Engineering, Hebei University of Technology, Tianjin, China; ^4^ School of Engineering, Saveetha University, Chennai, India; ^5^ Materials Program, Federal University of Piaui, Teresina, Brazil

**Keywords:** agricultural waste, biochar, dyes, heavy metals, pesticides, pharmaceutical products, polycyclic aromatic hydrocarbons

## Abstract

The rapid growth of various industries has led to a significant, alarming increase in recalcitrant pollutants in the environment. Hazardous dyes, heavy metals, pesticides, pharmaceutical products, and other associated polycyclic aromatic hydrocarbons (such as acenaphthene, fluorene, fluoranthene, phenanthrene, and pyrene) have posed a significant threat to the surroundings due to their refractory nature. Although activated carbon has been reported to be an adsorbent for removing contaminants from wastewater, it has its limitations. Hence, this review provides an elaborate account of converting agricultural waste into biochar with nanotextured surfaces that can serve as low-cost adsorbents with promising pollutant-removing properties. A detailed mechanism rationalized that this strategy involves the conversion of agrowaste to promising adsorbents that can be reduced, reused, and recycled. The potential of biowaste-derived biochar can be exploited for developing biofuel for renewable energy and also for improving soil fertility. This strategy can provide a solution to control greenhouse gas emissions by preventing the open burning of agricultural residues in fields. Furthermore, this serves a dual purpose for environmental remediation as well as effective management of agricultural waste rich in both organic and inorganic components that are generated during various agricultural operations. In this manner, this review provides recent advances in the use of agrowaste-generated biochar for cleaning the environment.

## 1 Introduction

Biomass from plants, animals, and microbes are thermally decomposed without any oxygen supply to produce a porous carbonaceous material termed as biochar. The nanotextured surfaces of biochar can be used for the removal of hazardous dyes, heavy metals, pharmaceutical waste, and other toxic pollutants due to their superior adsorbing properties (Nidheesh et al., 2021; Sakhiya et al., 2020; Enaime et al., 2020). Biochar also has tremendous applications in catalysis, energy storage and supercapacitors, carbon sequestration, and animal feed (Esteves et al., 2020; Senthil and Lee, 2021; Jones et al., 2018; Chiappero et al., 2020). However, it is important to note that the physico-chemical properties of biochar are largely dependent on the type of raw material used for the production of that biochar. Other important key determinants for the application of biochar depend on the temperature, duration of heating, type of gas flow, catalysts used, pH, porosity, surface area, and moisture content (Amalina et al., 2022a; Kazemi et al., 2020; Li et al., 2019a). The chemical composition of the biomass (such as cellulose, hemicellulose, and lignin) also influences the physico-chemical properties of the biochar (Liu et al., 2018; Singh et al., 2020). The agricultural waste and plant materials used during biochar production are mainly considered as lignocellulosic biomass (Krishnan et al., 2021). Low concentrations of heavy metals and heteroatoms (such as nitrogen, phosphorus, and sulfur) make lignocellulosic biomass less harmful and the most preferred raw material for biochar production (Senthil and Lee, 2021). Biochar is also used for the improvement of soil quality. Furthermore, the high carbon content of biochar rationalizes it as an efficient fuel for power generation (Daful and Chandraratne, 2018).

Agrowastes and stubbles can serve as a cheap source of carbonaceous biomass that can be subjected to thermo-chemical conversion at temperatures as high as 300°C–900°C under oxygen-limited conditions. These processes may include gasification, hydrothermal carbonization, pyrolysis, and torrefaction (Nidheesh et al., 2021). Cellulose is the most predominant biomolecule in plant biomass as it is abundantly present in the cell wall of plants, while hemicellulose and lignin are the second and third highly prevalent polymers, respectively (Singh et al., 2020). During pyrolysis, cellulose and hemicellulose are decomposed faster than lignin, while other inorganic components are converted to ash (Li et al., 2020). The non-structural support in the biomass, usually referred to as “organic extractives,” includes alkaloids, fatty acids, glycosides, gums, mucilages, pectins, phenolics, proteins, resins, saponins, simple sugars, starch, terpenes, and waxes (Guo et al., 2018; Nyoo et al., 2021; Singh and Chandra, 2019). Hence, agrowaste-based biochar production is more advantageous as it provides attractive properties, such as more surface area, high cation exchange property, stability, and porosity.

This process is not only simple and efficient but also environmentally benign, recyclable, economical, and rapid (Amalina et al. 2022b). Amide, amine, carboxylic (COOH), hydroxyl (OH), and lactonic functional groups attached to the surface of the biochar play a critical role in determining its ability to remove various pollutants by adsorption (Yaashikaa et al., 2020). Furthermore, the efficiency of biochar in environmental purification can be enhanced by pre-treatment and post-treatment of biochar using appropriate acidic, alkali, and/or oxidizing agents (Xiang et al., 2020; Wahi et al., 2017). Various analytical techniques, such as scanning electron microscopy (SEM) and transmission electron microscopy (TEM), are used for morphological evaluation. These techniques are efficient in determining the microtopography of the biochar surfaces. Fourier-transform infrared spectroscopy (FTIR) helps examine the surface-associated functional chemical groups, while the Brunauer–Emmett–Teller (BET) method plays an important role in evaluating the surface area and porosity. Thermogravimetric analysis (TGA) can determine the pollutant percentage loading onto the biochar surface, while nuclear magnetic resonance (NMR), Raman spectroscopy, and X-ray diffraction (XRD) are employed to determine the structure and elemental composition of the biochar (Sonu et al., 2020; Zhang et al., 2020).

In view of this background, this review provides a comprehensive account on the recent advances on agrowaste-based biochars with respect to their production parameters, physico-chemical properties, and applications in environmental remediation.

## 2 Environmental remediation

Several researchers have attempted to use agrowaste-based biochars as a superior adsorbent for wastewater treatment (Decima et al. 2021). Their large surface area, aromatic and activated carbons, high porosity, and high specific surface area have rendered them with a high capacity to adsorb refractory pollutants, such as hazardous dyes, heavy metals, pesticides, pharmaceutical products, and polycyclic aromatic hydrocarbons, that are discussed in the subsequent sections (Legan et al. 2022; Xiao, 2022).

### 2.1 Hazardous dyes

Several industries use numerous hazardous synthetic dyes that are highly toxic to aquatic life, animals, and plants. The conventional methods used for treating dye-contaminated industrial effluent include membrane separation, photochemical and electrochemical oxidation, ion exchange, Fenton’s process, and coagulation (Karmakar et al. 2021; Bloch et al. 2021; Nandre et al. 2021). However, these methods are often insufficient and ineffective depending on the type of dye contaminant and the volume of the effluent to be treated. Moreover, they generate a large amount of sludge and toxic intermediates (Ghosh and Webster, 2021; Ghosh et al. 2022a; Ghosh and Sarkar, 2022). Thus, agrowaste-based biochar-mediated adsorptive removal of dyes has drawn more attention due to its ecofriendly nature, high selectivity, and low cost of operation, as shown in Table 1.

**TABLE 1:** Agrowaste-generated biochar for dye-removal applications.

Biochar source	Dye	pH	Temperature (°C)	Time	Removal efficiency	Reference
Bael shell (*Aegle marmelos*)	Patent Blue (V)	2.7	30 ± 5	0–1 h	78%	Roy et al. (2018)
Banana peel	Reactive Black 5	3	55	24 h	97%	Kapoor et al. (2022)
Orange peel	Congo red	2–3	-	24 h	93% (MAB–steam), 89% (MAB–CO_2_)	Yek et al. (2020)
Rice husk and bamboo	Basic Red 46	9–11	35	24 h	9.06 mg/g (bamboo) and 22.12 mg/g (rice husk)	Sackey et al. (2021)
Rice husk	Methylene blue	7 ± 1	27.5 ± 1	60–72 h	71%–99%	Ahmad et al. (2020)
Rice husk	Reactive Red 120	2	35	-	97.5%	Krishnasamy et al. (2022)
Soybean straw	Brilliant green	8	60	60 min	99.73%	Vyavahare et al. (2021)
Sugarcane bagasse	Methylene blue and crystal violet	11	25–65	15 min	≥98%	Moharm et al. (2022)
Green tea waste	Methylene blue	5	-	40 min	98.07%	El-Azazy et al. (2021)

Roy et al. (2018) developed biochar from bael shell (*Aegle marmelos*) for the removal of Patent Blue (PB) (V) dye from an aqueous solution. The bael shell biochar (BSB) was prepared by first washing and drying the bael shell in sunlight for 10 days. Then, the dehydrated bael shell was crushed and ground into a fine powder containing 250-µm particles that was carbonized in a pyrolyzer at 500°C for 3 h, followed by cleaning with distilled water and drying at 75°C for 2 h. The point of zero charges (ΔpH_z_) for BSB was 8.80. The change in the surface morphology of BSB before and after adsorption was analyzed by SEM. After adsorption, peaks shifted from 2,523.7 to 2,463.9 cm^−1^, which indicated the interaction of the O–H bond of the carboxylic acid group with the dye, while the peak at 1,308.1 cm^−1^ was attributed to –SO_3_^−^ that was considered the main functional group for PB (V) dye interaction on the BSB surface, as indicated by FTIR. A decrease in the PB (V) removal efficiency was noted with an increase in pH. Maximum dye adsorption was observed at pH 2.7 at an efficiency of 74% (3.7 mg/g) at 30°C ± 5°C when kept in contact for 0–1 h. A pseudo-second-order model showed a better description of the sorption on BSB. The regression coefficients (*R* ^2^) calculated from the Freundlich model (0.968) were higher than those obtained from the Langmuir model (0.4224), indicating the dominance of the multilayer sorption process. Van der Waals forces, hydrogen bonding, and electrostatic interactions might be the underlying mechanism behind the sorption of PB (V) by BSB. The carboxylic group present on the BSB surface was completely dissociated at higher pH, which created repulsion between the carboxylate ion of BSB and the negatively charged portion of the PV dye, resulting in the removal of PV (V) dye from wastewater. BSB (carboxylic group) provides a positively charged surface with electrostatic interrelationships to the –SO_3_^−^ group of the PB (V) dye. The van der Waals forces play a role due to the hydrophobic region of BSB and hydrophilic region of the PV (V) dye and protonation of carboxylic and hydroxyl groups at lower pH.

Kapoor et al. (2022) generated banana peel biochar (BPB) for the removal of Reactive Black 5 (RB5) dye. The BPB was prepared by pyrolyzing dried banana peels at 500°C for 3 h. The rough and irregular surface morphology of BPB after the adsorption of RB5 was evident from the SEM analysis. The absorbance peaks of C–H (2,920 and 2,913 cm^−1^), C=C (2,259 cm^−1^), and C=O (1,705 and 1,726 cm^−1^) were noted in the FTIR spectra. The hydroxyl and carboxylic groups played an important role in adsorption. The highest dye removal of 96% was obtained at pH 3, while the highest adsorption of 97% was noted for 0.8 g of BPB. A reduction in the size of BPB increased the adsorption of the dye due to the large surface area of BPB. The equilibrium point of adsorption was 120 min. Higher removal efficiency was achieved at a lower dye concentration. Maximum adsorption (97%) of the dye was obtained at 55°C. The isotherm studies revealed that the Langmuir model (*R* ^2^ = 0.948) was the best fit compared to the Freundlich model (*R* ^2^ = 0.4471), which followed monolayer adsorption. The kinetic studies showed that adsorption followed the pseudo-second-order model and chemisorption mechanism. The thermodynamic analysis identified negative ΔG° and ΔH° values, indicating that adsorption was spontaneous and exothermic in nature. Even after five cycles of adsorption–desorption, the BPB retained greater than a 38% sorption potential for RB5 dye, indicating high regeneration efficiency.

Yek et al. (2020) developed biochar from orange peel waste (OPW) by a single-step pyrolysis process by combining microwave heat and CO_2_ or steam activation to produce microwave-activated biochar (MAB). The OPW was heated at 100 °C in an open-top quartz funnel, following CO_2_ or steam injection for 15 min at 100°C–700°C at a constant rate of 5 L/min, which increased the pore size and provided a large number of adsorption sites. BET analysis showed that the surface area of the biochar increased from 95.6 m^2^/g to 305.1 m^2^/g (MAB–steam) and 158.5 m^2^/g (MAB–CO_2_), which indicated that using CO_2_ and steam as activating agents increased the surface area and pore size of the biochar. A high mass yield (31–44 wt%), high content of fixed carbon (58.6–61.2 wt%), and low ratio of H/C (0.3) and O/C (0.2) were noted for the MAB. SEM images showed a coarse and less-porous surface of the OPW, while a more porous structure after CO_2_ activation was noted. CO_2_ and steam played a dual role in microwave-assisted pyrolysis. They acted as a purging gas to provide an inert environment for pyrolysis. Furthermore, they serve as activating agents for generating pores in the biochar by removing carbon atoms. The mechanism behind the formation of the pores was described as gasification, the Boudouard reaction, and the water–gas shift reaction. The CO_2_ and steam were gasification agents that penetrated the interior of the carbon material to increase porosity. Both CO_2_ and steam activations were endothermic reactions. Microwave pyrolysis was a rapid, targeted, and low-cost approach compared to conventional methods. The adsorption efficiency of MAB for Congo red (CR) was affected at pH above 3. Therefore, dye removal was accomplished under acidic conditions (pH 2–3). The maximum dye removal obtained by MAB–steam (136 mg/g) was approximately 93% compared to that obtained by MAB–CO_2_ (91 mg/g), which was approximately 89% with a biochar dosage of 0.3 g/100 mL and a contact time of 24 h. Kinetic studies showed that the MAB–CO_2_ and MAB–steam followed the pseudo-second-order model. The adsorption isotherm for MAB–CO_2_ was well fit with the Langmuir model (*R* ^2^ = 0.95), and MAB–steam with the Freundlich model (*R* ^2^ = 0.879). The mechanism behind the adsorption of CR dye by MAB was mainly due to the electrostatic interaction of the adsorbent, and the acidic condition significantly enhanced the attractive force between the adsorbent and anionic dye (CR). The sulfonic group (–SO_3_^−^) of the dye was also responsible for adsorption.

Sackey et al. (2021) developed bamboo and rice husk biochar for the removal of the Basic Red 46 (BR46). The biochar was produced by pyrolyzing biomass under an oxygen-limited condition at 500°C for 6 h at a heating rate of 10°C min^−1^. The obtained biochar was ground and passed through 100-mesh sieves and denoted as B500 (bamboo) and R500 (rice husk). BET analysis indicated the surface area of B500 and R500 to be 1.99 m^2^/g and 5.21 m^2^/g, respectively. The average pore volume for B500 and R500 was 0.004 cm^3^/g and 0.012 cm^3^/g, respectively. SEM images showed a thick and smaller pore volume for B500 and a thin and large pore volume for R500. XRD analysis identified that the amorphous pore structure of B500 and R500 was due to the presence of lignin, hemicellulose, and silicate. FTIR analysis revealed that the Si–O–Si group was responsible for the adsorption of BR46, and the band observed at 471 cm^−1^ was attributed to Si content. It was noted that the R500 biochar had high Si content and an O-containing functional group compared to B500. The adsorption efficiency increased at pH ranging from 9 to 11. The adsorption efficiency of BR46 increased with an increase in the temperature. The adsorption efficiencies of B500 and R500 were 9.06 mg/g and 22.12 mg/g, respectively, at 35°C with a contact time of 24 h. The results indicated higher adsorption efficiency of R500 compared to B500. A kinetic study revealed that the pseudo-second-order (*R* ^2^ ˃ 0.99) model was the best fit for adsorption. The adsorption isotherm results showed the Langmuir model (*R* ^2^ = 0.99) to be the best fit compared to the Freundlich model (*R* ^2^ = 0.898–0.958), which followed monolayer adsorption. A thermodynamic study identified negative ΔG° values that indicated a more spontaneous adsorption process, while the ΔH° values indicated its endothermic nature at a higher temperature. The K_d_ value of B500 and R500 ranged from 10^2^–10^3^. The π–π interaction, cation exchange, and electrostatic interaction might be the underlying mechanisms behind the sorption of BR46 by B500 and R500. BR46 can act as an electron acceptor, which contributed to the n–π electron donor–acceptor (EDA) during adsorption.

Ahmad et al. (2020) developed a rice husk-derived biochar for the removal of a methylene blue (MB) dye. The RHB was produced by unconnected pyrolysis for 3 h at 500°C at a heating rate of 5°C/min. The pH of the RHB was 7.68, while the yield and total organic carbon were 258 g/kg and 53.6 g/kg, respectively. FTIR analysis showed absorbance peaks for H, O–H, C=N, C–O, Si–O–Si, and C=C. SEM images confirmed a thick surface with a porous structure for RHB. The adsorption efficiency of RHB for MB was 71%–99% at pH 7 ± 1°C and 27.5°C ± 1°C. The dye removal efficiency was 95.9% when the contact time was 60–72 h. It was noted that an increase in the contact time to 96 h resulted in a decrease in dye adsorption. Maximum dye removal up to 95.9% was achieved with a biochar dosage of 0.5 g/100 mL. Kinetic studies indicated that the adsorption isotherm followed the Freundlich isotherm model (*R* ^2^ = 0.952) and the pseudo-second-order model (*R* ^2^ = 0.998).

Krishnasamy et al. (2022) developed agricultural waste-derived biochar from rice husks (the raw material) for the remediation of Reactive Red 120 (RR120) from an aqueous solution. The raw material was washed and dried for 24 h and was then pulverized into a fine powder containing 75-mm particles. The biochar was produced by pyrolyzing the raw material in electrical muffle furnaces for 120 min at 400°C and was allowed to cool down under normal conditions. TGA revealed that the highest decomposition of the rice husk was at 100°C–350°C with a total weight loss of 34.21%. SEM identified the porous surface structure before adsorption, which was converted to a smooth surface after adsorption. FTIR analysis revealed significant absorbance peaks before adsorption for =C–H (792.73 cm^−1^), C–O (1,071.09 cm^−1^), C=C and N–H (1,507.30 cm^−1^), O–H and N–H (3,309.26 cm^−1^), C–H (2,921.04 cm^−1^), and C≡C bonds (2,110.44 cm^−1^). FTIR peaks after adsorption of RR120 showed shifts in =C–H (791.49 cm^−1^), C–O (1,034.52 cm^−1^), C=C and N–H (1,508.92 cm^−1^), O–H and N–H (3,316.06 cm^−1^), C–H (2,921.78 cm^−1^), and C≡C bonds (2,107.46 cm^−1^). An increase in the biochar dosage from 0.5 to 10 g/L resulted in a decrease in the removal efficiency and uptake capacity of RR120. The optimal dosage of biochar was taken as 1 g/L. The highest sorption ability was observed at pH 2 due to electrostatic interactions between anions of the biochar and dye. The removal efficiency increased upon a decrease in temperature. At 35°C (the optimal temperature), the removal efficiency of RR120 was approximately 56.13%. Increasing the concentration of the dye showed no change in the uptake capacity, so the optimal initial dye concentration was taken as 15 mg/L. A maximum desorption efficiency of 97.5% was attained by sodium hydroxide in three sequential sorption–elution cycles.

Vyavahare et al. (2021) produced biochar from soybean straw biomass (SSB). The biochar was prepared by pyrolyzing the SSB in tubular muffle furnaces at different temperatures (400, 600, 800, and 1,000°C) for 1 h under oxygen-limiting conditions. SEM images showed a rough surface morphology of SSB, which was converted to a smooth surface after brilliant green dye (BG) adsorption. BET analysis determined the surface area of SSB to be 194.7 m^2^/g, while XRD identified the formation of a crystalline structure with an average size of 29.53 nm. The devolatilization reaction of SSB started at 400°C, which was identified by TGA. FTIR analysis identified absorbance peaks for C–H (2,884.82 cm^−1^), C–C (1,401.87 cm^−1^), C–N (1,242.05 cm^−1^), O–H (3,251.46 cm^−1^), and S=O bonds (1,034.59 cm^−1^). Maximum dye removal was obtained at 800°C. The dye removal increased by increasing the dose of SSB (0.4 g), BG (3,000 mg L^−1^), and temperature (60°C). Higher sorption was achieved at an initial contact time of 60 min (92.43%). It was noted that the dye sorption increased at pH 8 (74.79 mg g^−1^ and 99.73%). Kinetic studies revealed that the pseudo-second-order model was a better fit for sorption (*R* ^2^ = 0.997–0.998) than the pseudo-first-ordered model (*R* ^2^ = 0.761–0.885). The regression coefficients (*R* ^2^) calculated from the Freundlich model (0.990–0.996) were higher than those calculated from the Langmuir model (0.953–0.981), indicating the dominance of the multilayer sorption process. Thermodynamic studies found that adsorption was spontaneous, endothermic, and initiated by dipole–dipole interactions.

**FIGURE 1:**
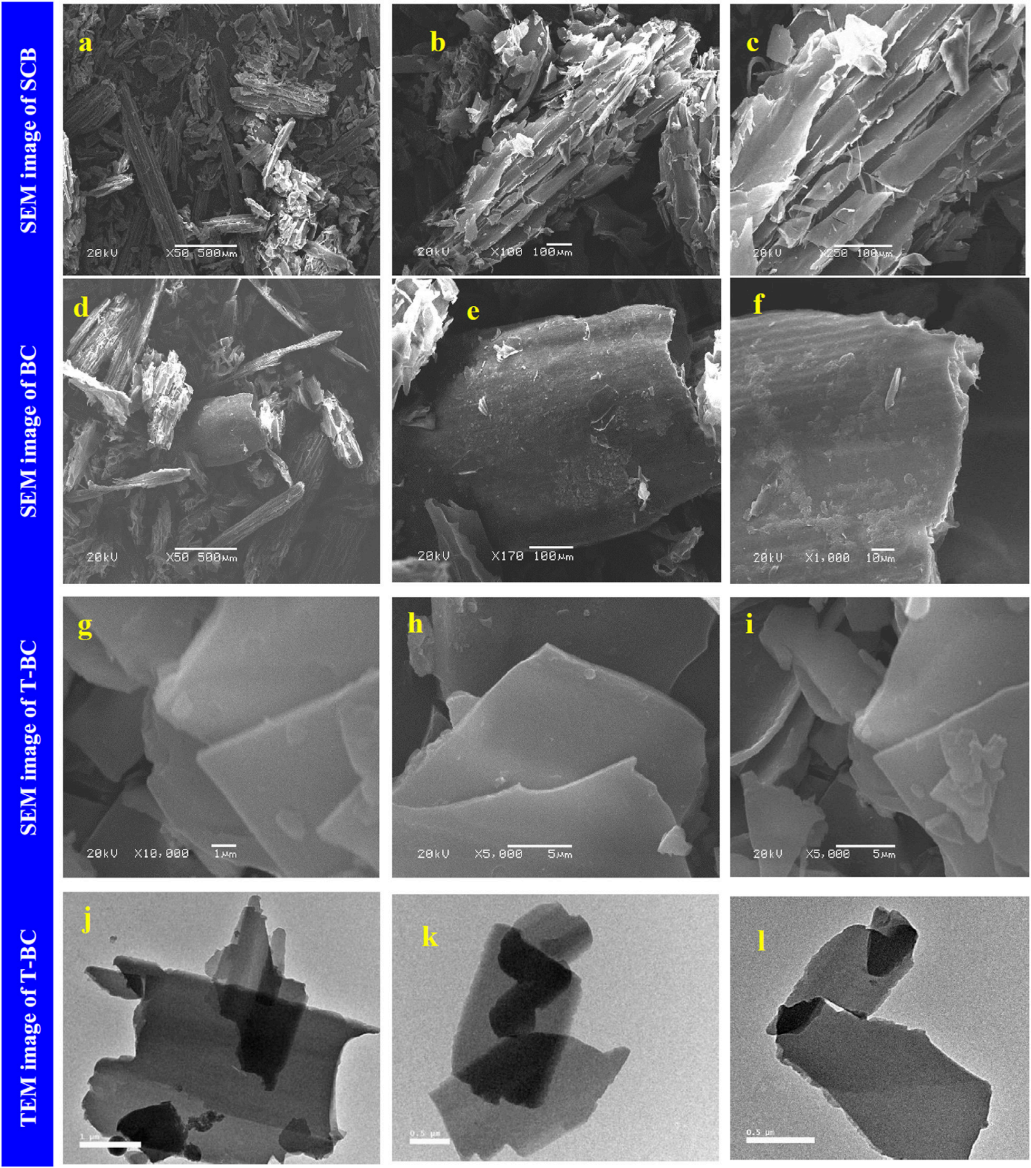
SEM image of SCB **(A–C)**, BC **(D–F)**, and T-BC **(G–I)**, and TEM image of T-BC **(J–L)**, reprinted from the work of Moharm, A. E., El Naeem, G. A., Soliman, H. M., Abd-Elhamid, A. I., El-Bardan, A. A., Kassem, T. S., Brase, S. (2022). Fabrication and characterization of effective biochar biosorbent derived from agricultural waste to remove cationic dyes from wastewater. Polymers 14(13), 2,587.

Moharm et al. (2022) developed biochar from sugarcane bagasse for the removal of cationic dyes from wastewater. The biochar was prepared by pyrolyzing 1 g of dry bagasse powder in a muffle furnace at 350°C for 30 min at a heating rate of 10°C/min. Furthermore, the black powder (BC) (0.7 g) formed was treated with different NaOH (W/V) concentrations (1%, 2%, 5%, and 10%) for 24 h on a magnetic stirrer to provide biochar denoted as T-BC-1, T-BC-2, T-BC-5, and T-BC-10, respectively. The smooth-layered structure with a flat surface and parallel sheaths of BC was converted into a smoother surface after treatment with NaOH, as observed in the SEM images shown in Figure 1. The absorption peaks at 3,480 and 3,392 cm^−1^ were attributed to the presence of methylene blue (MB) and crystal violet (CV), respectively, which was analyzed by FTIR. Raman spectra analyzed the G-band at 1,583 cm^−1^, which was attributed to the sp^3^ hybridization of a carbon atom, and the D-band at 1,353 cm^−1^, which was attributed to defects in sp^2^ hybrid carbon and heteroatoms. BET analysis confirmed that T-BC had the highest surface area (8.2175 m^2^/g), pore volume (6.3102 cm^3^/g), and mesoporous structure with an average pore diameter of 30.716 nm due to the NaOH treatment. A highly organized nanosheet biochar was identified by XRD. Energy-dispersive X-ray (EDX) analysis showed the presence of Na atoms in T-BC after the BC was treated with NaOH. The highest adsorption efficiency was obtained for 1% NaOH-treated BC. The removal efficiency of MB and CV increased quickly in the first 15 min. Intra-particle diffusion played a significant role in absorption. A decrease in adsorption by increasing the dye concentration due to the limited number of adsorbent surface sites was prominent. The adsorption efficiency increased with an increase in the adsorbent dose up to 0.015 g (MB) and 0.025 g (CV). An increase in the removal efficiency by increasing temperature (25°C–65°C) was attributed to the increased porosity and total pore volume of the adsorbent. The highest adsorption efficiency was obtained at pH 11. The pseudo-second-order model was the best fit, while the adsorption isotherm followed the Langmuir model (*R* ^2^ = 0.996 (MB) and 0.9998 (CV)), which indicated the dominance of monolayer sorption. The maximum adsorption efficiency of MB and CV was 114.42 and 99.50 mgg^−1^ (≥98%), respectively. A thermodynamic study revealed a negative ΔG° value, indicating the exergonic and spontaneous nature of adsorption, while a positive ΔH° value indicated its endothermic nature. The hydrogen bonding, electrostatic interactions, and van der Waals forces might be the underlying mechanisms behind the sorption of MV and CV by T-BC.

### 2.2 Heavy metals

Heavy metals [such as lead (Pb), cadmium (Cd), and chromium (Cr)] are highly toxic in their ionic form, and can cause metabolic impairment in animals and plants after intake (Ghosh et al. 2021a; Ghosh et al. 2021b). Hence, various biological approaches are being developed for the bioconversion and bioremoval of these toxic pollutants, which include mechanisms like redox processes, adsorption, complexation, ion exchange, precipitation, and electrostatic attractions (Ghosh et al. 2022b). Biochar-mediated heavy metal removal seems to be a sustainable efficient approach, as shown in Table 2.

**TABLE 2:** Agrowaste-generated biochar for heavy metal-removal applications.

Biochar sources	Metals	pH	Temperature	Time	Removal efficiency	Reference
Avocado seed (AS), avocado peel (AP), and grapefruit peel (GP)	Pb^2+^	6.88–9.65	-	1 h	98%–99% (AST and GPT) and 97% (ASB)	Granados et al. (2022)
Banana peel	Cu^2+^ and Pb^2+^	5.5 (Cu^2+^) and 9 (Pb^2+^)	-	30 min	50%–70%	Amin et al. (2018)
Banana pith	As (V)	7.5	298 K	2 h	100%	Lata et al. (2019)
Corn stover (CS), orange peel (OP), and pistachio shell (PS)	Pb^2+^	6	25°C	1 h	94%	Mireles et al. (2019)
Date palm	Pb^2+^ and Cu^2+^	4.5 (Pb^2+^); 5.5 (Cu^2+^)	-	(Pb^2+^) (Cu^2+^)	98.9 mg g^−1^ (Pb^2+^) and 41 mg g^−1^ (Cu^2+^)	Amin et al. (2019)
Peanut husk	Cr (VI)	2	45°C	180 min	95.27%	Mahanty and Mondal. (2021)
Peanut shell	Pb^2+^, Cd^2+^, Ni^2+^, Cu^2+^, and Zn^2+^	5	25°C	-	239.2, 188.6, 119.96, 147.8, and 81.8 mg/g	Shan et al. (2020)
Rice straw	Cu (II)	-	-	180 min	28.88 mg g^−1^	Sakhiya et al. (2021)
Sugarcane bagasse	Pb(Ⅱ)	5	25°C	30 min (SC-BC) and 15 min (OP-BC)	80.22 mg/g (SC-BC) and 27.86 mg/g (OP-BC)	Abdelhafez and Li (2016)
Walnut shell	Cr (VI)	5.5	-	2	93%	Kokab et al. (2021)

Granados et al. (2022) developed biochar from avocado seed (AS), avocado peel (AP), and grapefruit peel (GP) for the removal of Pb^2+^ from an aqueous solution. The biochar was prepared using two different pyrolysis methods. The first group of biochar was prepared in a tube furnace by minimizing oxygen inflow in the presence of N_2_ flow at a heating rate of 5 L min^−1^. Furthermore, the biomass was pyrolyzed at 300 °C for 1 h to yield biochar denoted as AST, APT, and GPT. In the second group, the biochars were produced by BioCharlie Log by minimizing the oxygen inflow to yield biochar denoted ASB, APB, and GPB. The biochar yield from this slow pyrolysis method obtained from the tube furnace and BioCharlie was 42%–63% and 36%–51%, respectively. An increase in pyrolysis temperature resulted in a decrease in biochar yield. This is more significant and advantageous than the fast pyrolysis method. The pH of the biochars ranged from 6.88 to 9.65, which was dependent on the biomass type and biochar production method. This can determine the charge on the functional groups, which is crucial for binding of the heavy metal ions. The maximum adsorption efficiency was attained with a concentration of 10 mg/L of metal, 0.8 g of biochar, and 1 h equilibration time. The absorbance peaks of aromatic C–H (3,050 cm^−1^), aliphatic C–H (2,935 and 2,885 cm^−1^), C=O (1,740–1,700 cm^−1^), C=C (1,600 cm^−1^), and C–O–H or C–O–R (1,030 cm^−1^) were observed in the FTIR spectra for all of the biochars except APT. This shows the critical role of the functional groups in determining heavy metal binding efficiency. All the biochars showed greater than 90% Pb^2+^ removal efficiency, but the highest adsorption was obtained in AST and GPT, which was approximately 98%–99%. ASB showed the highest adsorption of 97%. The enhanced performance of AST and GPT might be attributed to their high ash content (approximately 68%), which is postulated to play a positive role in enhanced heavy metal adsorption.

Amin et al. (2018) developed biochar from a banana peel for the removal of copper (Cu^2+^) and lead (Pb^2+^). The biochar was prepared by pyrolyzing the biomass powder at 600°C for 3 h in a box furnace at a heating rate of 5°C/min. BET analysis determined the surface area (35.24 m^2^ g^−1^) of the banana peel biochar. BET analysis can determine specific surface areas and pore size distributions of biochar, and this highly efficient analytical technique is based on the physical adsorption of an inert gas, such as nitrogen, on the solid surface. Rough asymmetric pores were observed before adsorption, which were converted into a smooth and shiny closed pore structure after adsorption, as revealed by SEM. XRD analysis is advantageous as it is a non-destructive technique that can provide detailed information about the crystallographic structure, chemical composition, and physical properties of the biochar. The constructive interference of monochromatic X-rays and a crystalline biochar can provide valuable insights into the mechanism of heavy metal adsorption and the reactions taking place during the process. XRD analysis rationalized the fact that a few new compounds, such as cuprite, tolbachite, paramelaconite, and lead azide, were formed after the adsorption of Cu^2+^ and Pb^2+^, which suggested chemisorption or precipitation as the operating sorption mechanisms. FTIR analysis showed the absorbance peak of the C–H (600 and 700 cm^−1^) bond in aromatic and heteroaromatic compounds, while the peaks at 831 and 983 cm^−1^ were attributed to the C–H bond out-of-plane bending of an aromatic ring. The peak at 1,244 cm^−1^ represented the hydrogen-bonded hydroxyl compound, while the peak at 1,390 cm^−1^ represented CO_2_. The peak at 1,619 cm^−1^ was due to–COO– groups. The removal efficiency for both the heavy metal ions was approximately 40%–50% when pH was 3–9. The optimal pH value was 5.5 and 9 for Cu^2+^ and Pb^2+^ removal, respectively. This showed that heavy metal removal can actively occur in a wide range of pH values using the generated biochar. The equilibrium time was approximately 30 min. The removal efficiency increased with an increase in the adsorbent concentration for Cu^2+^ (1.4 g) and Pb^2+^ (1.8 g). The optimal Cu^2+^ and Pb^2+^ concentrations were 200 and 700 mg L^−1^, respectively. The Langmuir model (*R* ^2^ = 0.995) was well fitted for the adsorption of Cu^2+^. The Freundlich model (*R* ^2^ = 0.954) was the best fit for the adsorption of Pb^2+^. The adsorption isotherm followed a pseudo-second-order model for Cu^2+^ and Pb^2+^ adsorption. The integrated approach using FTIR, Langmuir, and Freundlich model fitting indicates the underlying mechanism of heavy metal adsorption. While FTIR emphasizes the role of the biochar surface-associated functional groups, the Langmuir and Freundlich models deciphered the kinetics of heavy metal adsorption.

Lata et al. (2019) produced iron-impregnated banana peel biochar (FeBPB) for the removal of As (V). The FeBPB was prepared by adding 100 mL of 250 g/L ferric nitrate nonahydrate solution to 4 g of banana pith followed by stirring at 500 rpm for 6 h, and the solution was left for 3 days in ambient conditions for proper iron distribution and successive diffusion in pores. Furthermore, the biochar was recovered by filtration and then washed and air-dried for 24 h. Field emission scanning electron microscopy (FESEM) images showed the porous surface morphology. After iron impregnation, the pores were filled with iron and the surface became rough. The specific surface area of FeBPB was 31.59 m^2^/g. FTIR analysis found absorbance peaks of FeBPB and FEAsBPB corresponding to –OH (3,386.29 shift to 3,383.65 cm^−1^) and C=O (1,027.81 shift to 1,082.69 cm^−1^) bonds. The shift indicates the hydroxyl and carbonyl groups involved in the adsorption of As (V). The peak at 690 cm^−1^ was attributed to As–O stretching. The amorphous structure was identified by XRD, while X-ray photoelectron spectroscopy (XPS) analysis revealed no conversion of As (V) to As (III). The highest removal efficiency (100%) was achieved at a very low concentration of FeBPB (2.5 g/L) compared to banana peel biochar (BPB). It was found that As (V) removal was approximately 91.05% at a contact time of 2 h. Higher removal of As (V) occurred at pH 7.5, with an effective As (V) concentration ranging from 10 μg/L to 150 μg/L. The thermodynamics study indicated an exothermic adsorption process. The maximum adsorption capacity of FeBPB at 298 K was 120.91 μg/g. Chemisorption with a pseudo-second-order model was a better fit mechanism than the pseudo-first-order model.

Mireles et al. (2019) developed biochar from corn stover (CS), orange peel (OP), and pistachio shell (PS) for Pb^2+^ removal from an aqueous solution. The biochar was produced by slowly pyrolyzing 20 g of biomass at three different temperatures (300, 450, and 600°C) for 1 h under N_2_ flow at a rate of 5 L min^−1^. The obtained biochars were denoted as CS300, CS450, CS600, OP300, OP450, OP600, PS300, PS450, and PS600. Biochar yield decreased with an increase in pyrolysis temperatures. The pH of the raw biomass was between 6.5 and 6.8 and showed a positive correlation with pyrolysis temperature. The highest surface area (279 m^2^/g) was observed for PS600 compared to OP300 (8.9 m^2^/g) and CS600 (3.6 m^2^/g). A decrease in the molar O/C ratios was noted at higher temperature (600°C). FTIR analysis identified amine, carboxyl, and hydroxyl groups to be responsible for the removal of Pb^2+^ in OP biochars, while carboxyl and hydroxyl groups played a role in CS and PS biochars. Amine was speculated to be an effective surface functional group for the removal of Pb^2+^. The effect of the pH of the solution was studied in the range from 2 to 6, and the best adsorption was observed at pH 6. The highest Pb^2+^ removal was obtained with CS600 and OP300, which was approximately >96% at pH 6. The two biochars, CS600 and OP300, showed more than 94% removal of Pb^2+^ at 10 mg/L. The adsorption equilibrium was observed in 1 h at 25°C. The Langmuir model (*R* ^2^ = 0.98) was a better fit than the Freundlich model (*R* ^2^ = 0.43–0.58), which indicated the dominance of monolayer sorption. The Pb^2+^ adsorption capacity was of the order CS600 (25 mg/g) > OP300 (11.111 mg/g) > PS600 (2.5 mg/g).

Amin et al. (2019) produced biochar from date palm biomass (DP_b_) for the removal of Pb^2+^ and Cu^2+^. The DP_b_ was prepared by pyrolyzing the biomass at 800°C for 3 h in a box furnace at a heating rate of 5°C/min. SEM images showed the formation of new channels and pores and white spots on the biochar surface, indicating the presence of smaller mineral particles on the biochar surface. XRD analysis determined the variation in minerals after the adsorption of Pb^2+^ and Cu^2+^. A few compounds, such as hydrocerussite (Pb_3_(CO_3_)_2_(OH)_2_), cerussite (PbCO_3_), and cuprous oxide were formed, which suggested chemisorption or precipitation as the operating sorption mechanisms. FTIR analysis showed that the absorbance peaks for –OH, –COOH, C–H, and Si–O bonds were lost after pyrolysis, while the C–O–C bond was observed in DP_b_ after pyrolysis. The equilibrium adsorption efficiency for Pb^2+^ and Cu^2+^ was achieved at 30–60 min and 2–3 h, respectively. The adsorption capacity for Pb^2+^ and Cu^2+^ was 98.9 mg g^−1^ and 41 mg g^−1^ at pH 4.5 and 5.5, respectively. The maximum removal of Pb^2+^ and Cu^2+^ up to 60% was obtained with DP_b_ doses equivalent to 1.0 and 1.8 g, respectively. The adsorption capacity of DP_b_ for Pb^2+^ and Cu^2+^ increased to 96 and 180 mg g^−1^, respectively, when the initial metal ion concentration increased from 50 to 250 mg L^−1^. The Freundlich–Langmuir and H–J isotherm models were well fitted to the adsorption of Pb^2+^ (*R* ^2^ = 0.95) and Cu^2+^ (*R* ^2^ = 0.92), respectively. A kinetics study showed that the adsorption isotherm followed the pseudo-second-order model (*R* ^2^ = 0.9999) for Pb^2+^ and Cu^2+^.

Mahanty and Mondal (2021) synthesized modified magnetic biochar (MBC_PH_) from peanut husk for the removal of Cr (VI) from an aqueous solution. The MBC_PH_ was synthesized by mixing 10 g of dried peanut husk with 80 mL of FeCl_3_.6H_2_O for 1 h, followed by stirring at 60°C for 0.5 h. Furthermore, pre-treated biochar was filtered (Whatman filter paper, grade 42) and dried at 70°C for 24 h. Then, the dried biomass was pyrolyzed in a tube furnace under a N_2_ atmosphere at 600°C for 2 h at a heating rate of 15°C/min. The standard biochar (BC_PH_) was prepared using the same procedure without being treated with a FeCl_3_.6H_2_O solution. After pyrolysis, an increased inorganic carbon content and decreased fixed carbon content of MBC_PH_ were noted due to the increasing iron content. An increase in pyrolysis temperature decreased the hydrogen, nitrogen, and oxygen content of MBC_PH_. BET analysis determined the surface area and pore volume of biochar, which increased from 16.48 to 183.62 m^2^/g and 0.132 to 0.263 cm^3^/g, respectively, after iron oxide modification. The abundant pores with diverse shapes and sizes were associated with the rough surfaces of BC_PH_, while a rugged surface structure was observed for MBC_PH_. XRD analysis confirmed that Fe_2_O_3_ was formed on the surface of MBC_PH_ since no peak was observed for amorphous carbon. FTIR analysis identified the absorbance peak of carboxyl and hydroxyl (3,384 cm^−1^), C=O (2,858 cm^−1^), C=C (1,628 cm^−1^), N–C=O (1,452 cm^−1^), –COOH (1,368 cm^-1^), CO^−^ (1,058 cm^−1^), phenolic group vibration (1,267 cm^−1^), and Fe–O bonds (582 cm^−1^). It was found that the PH_ZPC_ value obtained for MBC_PH_ and BC_PH_ was 2.65 and 6.5, respectively, which indicated that the MBC_PH_ was acidic in nature. The removal efficiency of Cr (VI) reached up to 95.27% for MBC_PH_ and 45.56% for BC_PH_ within 180 min. The maximum adsorption efficiency of Cr (VI) was obtained with an adsorbent dose of 0.2 g/50 mL and 180 min contact time at 45°C and pH 2. The Freundlich isotherm model was a better fit than the Langmuir and Temkin isotherm models for the adsorption of Cr (VI). The kinetics model described the adsorption isotherm following a pseudo-second-order model for both adsorbents BC_PH_ (*R* ^2^ = 0.0056) and MBC_PH_ (*R* ^2^ = 0.9998). A thermodynamic study identified that adsorption was spontaneous and the ΔG° value decreased with an increase in temperature. The ΔS° value was positive, indicating the randomness during adsorption, and the ΔH° value for both adsorbents indicated that adsorption was endothermic in nature. Adsorption for MBC_PH_ was controlled by the film diffusion process, and within 20 min, the removal efficiency reached 90%. The coexisting ions showed that there was minor interference of cations upon Cr (VI) removal, and the interference was directly proportional to the size of the cations or solubility of the electrolytes. It is important to note that a higher removal efficiency was retained up to the third regeneration cycle. The presence of HCO_3_^−^, SO_4_^2−^, and PO_4_^3−^ anions affected the removal capacity of MBC_PH_. The main mechanism behind the adsorption of Cr (VI) depends on the pH, point-zero charges of the synthesized material, and the electrostatic interactions between the adsorbent and CR (VI). Another mechanism was the presence of the O-containing group on the surface of MBC_PH_ and BC_PH_ that might facilitate the adsorption of Cr (VI).

Shan et al. (2020) synthesized biochar from peanut shells (PBC) for the removal of heavy metals in an aqueous solution. The PBC biochar was prepared by pyrolyzing the biomass in a tubular furnace in N_2_ flow at temperatures of 350, 400, 450, 500, and 600°C at a heating rate of 10°C/min for 2 h. The obtained biochar was denoted as PBC350∼PBC600. BET analysis indicated that the surface area and pore volume of biochar increased from 3.77 to 6.45 m^2^/g and from 0.0097 to 0.0161 cm^3^/g, respectively, with an increase in temperature from 350°C to 400°C. FTIR analysis revealed that the absorbance peak of –OH and C=C stretching and bending vibration decreased after adsorption, which indicated their involvement in adsorption. Higher surface area and pore volume were observed in PBC400. The pH value of the biochar increased from 9.11 to 10.35, and the biochar yield decreased from 47.9% to 33.6% with an increase in temperature from 350°C to 600°C. A decreased ratio of H/C (aromaticity), O/C (polarity), and (O+ N)/C (hydrophobicity) was noted with an increase in the pyrolysis temperature above 400°C. A smooth surface with small, hollow holes was observed before adsorption, and the rough surface morphology of biochar was observed after the adsorption of the metal ion. EDX analysis confirmed the elemental composition of PBC400 before adsorption, which included C, O, Cl, Na, K, Ca, and Mg, while after adsorption, the Cl, Na, K, and Ca were reduced with the simultaneous appearance of Pb. The higher removal capacity for the five heavy metals, Pb^2+^, Cd^2+^, Ni^2+^, Cu^2+^, and Zn^2+^, was 45.98, 34.22, 22.1, 33.5, and 15.39 mg/g, respectively. The best adsorption efficiency was obtained at pH 5 with an adsorbent dose of 20 mg. The Langmuir model (*R* ^2^ = 0.95) was a better fit than the Freundlich isotherm model for adsorption. The maximum adsorption capacity obtained by the Langmuir model for Pb^2+^, Cd^2+^, Ni^2+^, Cu^2+^, and Zn^2+^ at 25°C was 239.2, 188.6, 119.96, 147.8, and 81.8 mg/g, respectively. A kinetic study showed that the pseudo-second-order model (*R* ^2^ = 0.90) was the best fit for adsorption. The electrostatic interaction and O-containing group might play a significant role in the adsorption.

Sakhiya et al. (2021) produced biochar from rice straw (RBC) for the removal of Cu (II) in an aqueous solution. The RBC was prepared by pyrolyzing the biomass at different temperatures (300, 400, and 500°C) at a heating rate of 10°C/min. The inert N_2_ was supplied at a flow rate of 1.5 L min^−1^ for 1 h. The obtained biochar was named according to the pyrolysis temperature: RBC300, RBC400, and RBC500. The pH of the biochar increased from 7.24 to 11.24 by increasing the temperature from 300°C to 500°C. The pH of the rice straw was alkaline. The yield of biochar decreased from 57.87% to 35.63% upon increasing the pyrolysis temperature from 300°C to 500°C. An increased carbon content (68.72%–81.48%) and decreased oxygen (5.81%–2.34%) and hydrogen contents (56.38%–14.94%) upon increasing the pyrolysis temperature were noted. The H/C and O/C ratios decreased from 0.51 to 0.02 and 1.51 to 0.18, respectively, with an increase in pyrolysis temperature. BET analysis identified an increase in the specific surface area from 48.3 to 101.29 m^2^ g^−1^ upon increasing the temperature. SEM images showed more pore development in RBC400 and RBC500 than in RBC300. The highest adsorption efficiency was observed in RBC500 (28.88 mg g^−1^), and the lowest removal efficiency was observed in RBC300 (8.21 mg g^−1^). The maximum Cu (II) adsorption was achieved at an equilibrium contact time of 180 min at a metal ion concentration of 300 mg L^−1^. The adsorption isotherm followed a pseudo-second-order model. The Langmuir model (*R* ^2^ ˃ 0.99) was more suitable than the Freundlich (*R* ^2^ ˃ 0.95), which indicated the dominance of the monolayer sorption process.

Abdelhafez and Li (2016) developed biochar from sugarcane bagasse (SC-BC) and orange peel (OP-BC) to remove Pb(Ⅱ) ions from an aqueous solution. The biochar was produced by pyrolysis at a temperature of 500°C. The biochar was alkaline in nature, with an average pH of 9.63 (SC-BC) and 8.75 (OP-BC). SEM images showed the smooth surface of SC-BC and the uneven structure of OP-BC. BET analysis determined the high surface area of SC-BC (92.30 m^2^/g) compared to OP-BC (0.21 m^2^/g). FTIR analysis showed the absorbance peaks of C–OH (3,448 and 3,429.4 cm^−1^), C=O (1,637.27 cm^−1^), and C–C (1,384.85 cm^−1^). The maximum adsorption of Pb(Ⅱ) was attained at pH 5, contact times of 30 min for SC-BC and 15 min for OP-BC, 25°C, and a sorbent concentration of 1 g/L. The adsorption of Pb(Ⅱ) was an endothermic process. Pseudo-second-order kinetics (*R* ^2^ = >0.99) was followed. The adsorption isotherm studies revealed that the Langmuir model (*R* ^2^ = ≥0.97) was the best fit, with maximum adsorption capacities of 80.22 mg/g for SC-BC and 27.86 mg/g for OP-BC. Greater regeneration efficiency and desorption rates of Pb (II) ions were observed even after the fifth regeneration cycle.

**FIGURE 2:**
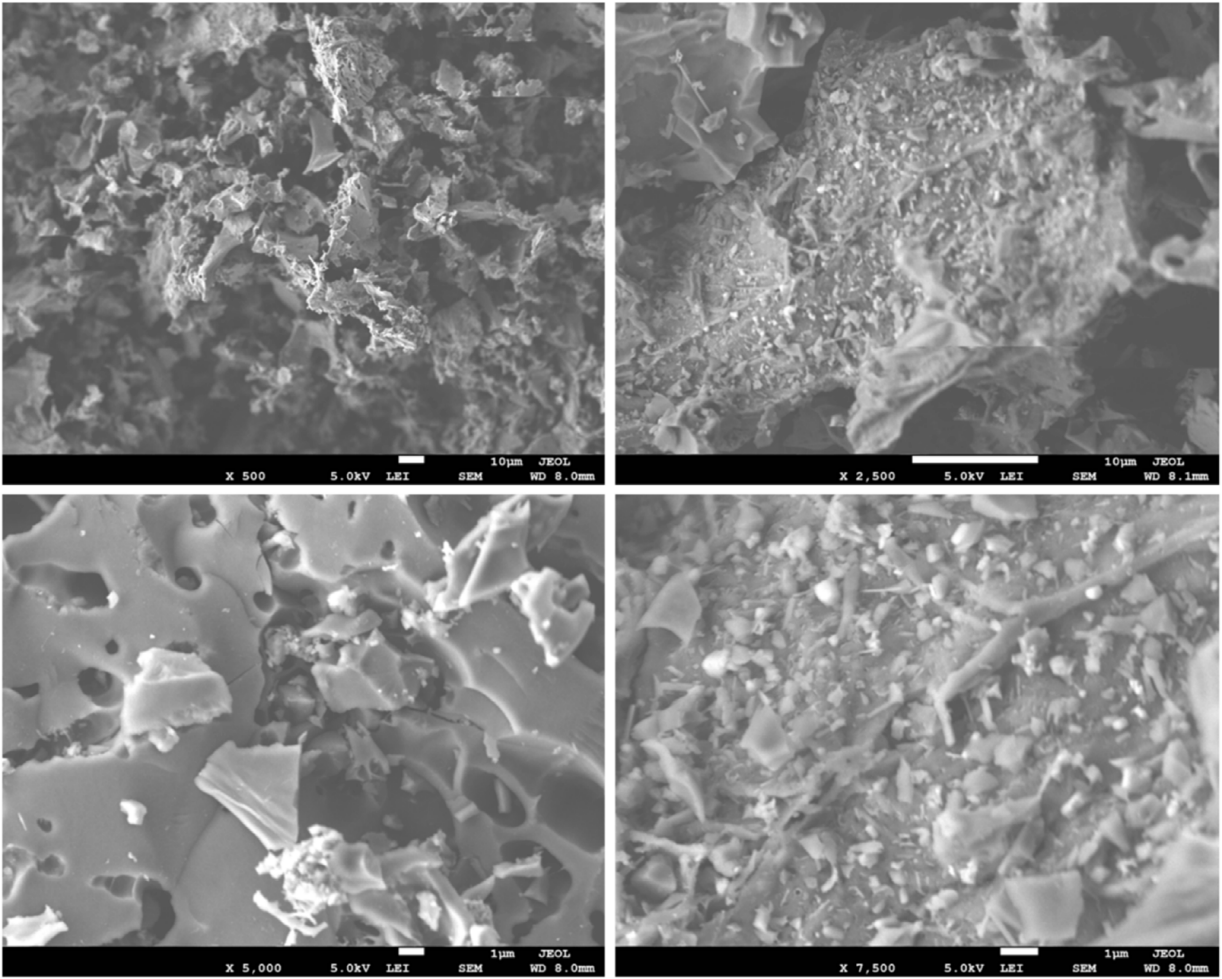
SEM micrographs of the walnut shell biochar, reprinted with permission from the work of Kokab, T., Ashraf, H.S., Shakoor, M.B., Jilani, A., Ahmad, S.R., Majid, M., Hakeem, K. R. (2021). Effective removal of Cr (Vi) from wastewater using biochar derived from walnut shell, Int. J. Environ. Res. Public. Health. 18(18), 9670.

Kokab et al. (2021) synthesized biochar from a walnut shell (WSB) for the removal of Cr (VI). The WSB was synthesized in a muffle furnace at 450°C for 2 h. SEM images showed the rough and uneven surface morphology with many pores, as shown in Figure 2. EDX analysis confirmed the presence of Cr (VI) in WSB. XPS results identified the presence of an O-containing functional group in the WSB that helped in the removal of Cr (VI). FTIR analysis showed the absorbance peaks of –OH (3,242 cm^−1^), C=O (1,695 cm^−1^), and C–H (875 cm^−1^), and the peak at 1,400 cm^−1^ represents carboxylates, quinones, and ketones. Maximum adsorption up to 93% was attained at a concentration of 110 mg L^−1^ Cr (VI) and 1.1 g L^−1^ WSB, with pH 5.5 and a contact time of 2 h. The correlation to the Langmuir isotherm model (*R* ^2^ = 0.89) was higher than that to the Freundlich model (*R* ^2^ = 0.89), which indicated the dominance of the monolayer sorption process. A kinetics study revealed that the pseudo-second-order model was best fit for adsorption.

### 2.3 Pesticides

The wide application of hazardous pesticides in various forms, such as granules, emulsions, wettable powder, and controlled-release polymers, has led to the contamination of both surface and ground water (Ghosh et al. 2022c). These toxic chemicals have detrimental effects on humans as they enter the food chain. Hence, various attempts are made to remove these pesticides using biochars given in Table 3.

**TABLE 3:** Agrowaste-generated biochar for pesticide removal applications.

Biochar sources	Pesticide	pH	Temperature (°C)	Time	Removal efficiency	Reference
Sugarcane bagasse	Chlorpyrifos	6	20–40	60 min	89%	Jacob et al. (2020)
Coconut shell	Diazinon	7	-	-	98.9%	Baharum et al. (2020)
Eucalyptus bark biochar (EBBC), corn cob biochar (CCBC), bamboo chip biochar (BCBC), rice husk biochar (RHBC), and rice straw biochar (RSBC) and acid-treated RSBC (T-RSBC)	Atrazine and imidacloprid	2–7.5	-	1 h	59.5%–89.8% (T-RSBC), 37.5%–70.7% (RSBC), 11.8%–42.6% (RHBC), 23.4%–40.1% (EBBC), 18.0%–30.4% (CCBC), and 12.3%–26.9% (BCBC) for atrazine	Mandal et al. (2017)
					58.2%–89.5% (T-RSBC), 39.9%–77.7% (RSBC), 28.0%–46.2% (RHBC), 16.9%–35.7% (BCBC), 14.7%–28.4% (EBBC), and 5.9%–20.1% (CCBC) for imidacloprid	
Rice husk	Carbofuran	5	-	-	160.77 mg g^-1^	Mayakaduwa et al. (2017)
Rice husk and tea refuse	Carbofuran	-	-	4 h	24.6 ± 0.4 mg/g (RHBC700) and 9.6 ± 0.2 mg/g (TWBC700)	Vithanage et al. (2016)
Sugarcane-filtered cake	Thiamethoxam	-	-	60 min	70%	Fernandes et al. (2021)
Tangerine seed-activated carbon	Carbamate pesticides (CMs)	7	20	15 min	Methiocarb (93.46 mg/g), carbaryl (44.64 mg/g), pirmicarb (39.37 mg/g), isoprocarb (13.95 mg/g), metolcarb (9.11 mg/g), and bendiocarb (7.97 mg/g)	Wang et al. (2020)

Jacob et al. (2020) studied the adsorption of chlorpyrifos (CPS) from aqueous solutions onto sugarcane bagasse biochar (SBC). The biochar (BC450) was produced by pyrolyzing the biomass in a muffle furnace at 450°C for 15 min. The BC was treated with acetone for 2 h to obtain biochar named AWC450, and the BC was treated with benzene for 2 h to obtain biochar named BWC450. BC450 showed a higher fixed carbon content compared to AWC450 and BWC450, which was attributed to the removal of the organic matter present in the bagasse by treatment with acetone and benzene. BC450 and AWC450 showed very high iodine and methylene blue values, which indicated the high adsorption capability of BC450 and AWC450. The point-zero charges determined the pH_ZPC_ for CPS (7.75), BC450 (7.8), AWC450 (7.1), and BWC450 (6.6). SEM images showed the small-sized pores and cavities of biochar. EDX analysis revealed that no other inorganic element was present in the biochar. FTIR revealed the peaks of C–H (749 and 849 cm^−1^), C–O and O–H (1,217 and 1,429 cm^−1^), C=C (1,521 cm^−1^), C=O (2,155 cm^−1^), C–H and OH (3,015 cm^−1^), and O–H (3,469 cm^−1^). XRD analysis confirmed the biochar to be amorphous. The maximum CPS adsorption attained in 60 min was approximately 89%, at pH 6, a CPS concentration of 10 ppm, 0.5 g of adsorbent, and temperature between 20°C and 40°C. A thermodynamics study revealed negative ΔG° and ΔH° values, indicating that adsorption was exothermic in nature, and the positive ΔS° value indicated randomness. The Freundlich isotherm model (*R* ^2^ = 0.9474) was a better fit than the Langmuir model. A kinetic study showed that adsorption followed the intra-particle diffusion model.

Baharum et al. (2020) studied the adsorption of diazinon from aqueous solutions onto coconut shell-modified biochar (BC). The carbonized coconut shell biochar (BC1), BC (BC2) was activated by slow pyrolysis using an electrical muffle furnace. The biomass was pyrolyzed at 700°C ± 35°C at a heating rate of 7°C/min for 2 h. Furthermore, chemical modification was carried out with acidic- and alkaline-activating agents using 1.0 M phosphoric acid (BC3) and 1.0 M sodium hydroxide (BC4), respectively. 0.5 g adsorbent was heated with 200 mL of chemicals at 80°C for 1 h. BET analysis determined a high surface area (405.97–508.07 m^2^/g) following the order BC3 > BC2 > BC4. SEM showed a highly porous surface with internal and external pores and regular structure. The EDX results confirmed the reduction of carbon and the increase in oxygen and potassium after adsorption, apart from the presence of the phosphorous compound in BC2. The highest removal of diazinon up to 98.9% was obtained at an optimal pH of 7 and BC3 dose of 5.0 g/L. The adsorption isotherm showed that BC1 (*R* ^2^ = 0.9971) and BC2 (*R* ^2^ = 0.9999) were best fit with the Freundlich isotherm model, indicating multilayer sorption onto the heterogeneous surface. The Langmuir isotherm model was a better fit for BC3 and BC4, which indicated monolayer adsorption onto the homogenous surface. The maximum adsorption capacity of BC3 was 10.33 mg/g, while that of BC2 and BC4 was 9.65 mg/g and 1.73 mg/g, respectively. BC2 and BC3 were highly efficient adsorbents with great diazinon-removing potential.

Mandal et al. (2017) prepared eucalyptus bark biochar (EBBC), corn cob biochar (CCBC), bamboo chip biochar (BCBC), rice husk biochar (RHBC), rice straw biochar (RSBC), and acid-treated RSBC (T-RSBC) for the adsorption of atrazine and imidacloprid from aqueous solutions. The biochars were prepared by pyrolyzing the biomass at 600 °C for 1 h. The T-RSBC was obtained by modifying the RSBC with H_3_PO_4_. The yield of the biochars was 28%–29% for BCBC, CCBC, and EBBC, and 35.2%–38.3% for RSBC and RHBC. The pH of CCBC was the highest (pH 10.1), while that of RSBC was the lowest (pH 8.8). The H_3_PO_4_-treated RSBC showed a pH of 6.9. The surface area of BCBC was larger (246.7 m^2^/g) than that of RHBC (159.1 m^2^/g), while the pore volume of RSBC was greater (6.455 cm^3^/g) than that of RHBC (0.018 cm^3^/g). The H_3_PO_4_-treated RSBC showed a reduced surface area of 192.3 m^2^/g and pore volume of 0.161 cm^3^/g compared to RSBC. The zeta potential of the biochars varied between 1.45 and −42.8 mV when studied in the pH range of 2–7.5. SEM images showed the porous structure of all the biochars containing repeated units of the channels. The adsorption kinetics indicated that the Elovich model was a better fit for the sorption of atrazine and imidacloprid. The fastest adsorption of atrazine and imidacloprid was obtained within 1 h by T-RSBC (58.9%–82.9%) compared to RSBC (28.7%–56.5%) and RHBC (16.3%–17.8%). The order of atrazine adsorption by the biochars was RSBC (37.5%–70.7%) > RHBC (11.8%–42.6%) > EBBC (23.4%–40.1%) > CCBC (18.0%–30.4%) >BCBC (12.3%–26.9%). Likewise, the order for imidacloprid adsorption was RSBC (39.9%–77.7%) > RHBC (28.0%–46.2%) > BCBC (16.9%–35.7%) > EBBC (14.7%–28.4%) > CCBC (5.9%–20.1%). The adsorption process followed the Freundlich isotherm model, which indicated the dominance of the multilayer sorption process.

Mayakaduwa et al. (2017) synthesized biochar from rice husk (RHB) for the removal of carbofuran in an aqueous solution. The biochar was prepared by pyrolyzing the biomass at 300, 500, and 700°C for 3 h at a heating rate of 7°C min^−1^, named RHBC300, RHBC500, and RHBC700, respectively. Steam activation of RHB700 was carried out at 5 mL min^−1^ for 45 min to obtain the biochar named RHBC700S. The yield of biochar decreased from 52% to 33% when temperature was increased from 300°C to 700°C. The oxygen, hydrogen content, and H/C ratio decreased with an increase in pyrolysis temperature. BET analysis determined the higher specific surface area and pore volume in RHBC700S to be 251.47 and 0.083 cm^3^ g^−1^, respectively. FTIR analysis revealed the peak of the OH bond (3,420 cm^−1^), which shifted to 3,440 cm^−1^, indicating the involvement of alcohol, phenols, and carboxylic acid groups in carbofuran adsorption via hydrogen bonding and dipole–dipole interactions. The peak at 1,630 cm^−1^ represented H–O–H bending after carbofuran adsorption by RHBC. The characteristic peak centered at 739 cm^−1^ in bare RHBC, which split into two new peaks at 754 and 719 cm^−1^ after the adsorption of carbofuran, indicated the π–π electron donor–acceptor interaction between the adsorbent and pesticide. The maximum adsorption was achieved at pH 5. The isotherm study showed that the Langmuir model was the best fit, with the maximum adsorption capacity of RHBC700S observed to be 160.77 mg g^−1^.

Vithanage et al. (2016) developed biochar from rice husk (RHBC) and tea refuse (TWBC) for the removal of carbofuran from aqueous solutions. The biochar was prepared by slow pyrolysis at 700 °C under oxygen-limited conditions for 3 h. The obtained biochar was labeled as TWBC700 and RHBC700. The biochar was alkaline in nature with pH of 10.21 (TWBC700) and 9.87 (RHBC). SEM revealed the porous surface morphology. The pore diameter of the surface was 1.75 nm (TWBC700) and 5.29 nm (RHBC). Thermodynamic studies revealed that the adsorption was spontaneous and exothermic with high randomness. The adsorption was fast in the first 2 h of contact time and then slowed down after 4 h of contact time, providing the highest adsorption of 24.6 ± 0.4 (RHBC700) and 9.6 ± 0.2 mg/g (TWBC700). The adsorption isotherm followed the pseudo-second-order model, with an adsorption of 25.2 and 10.2 mg/g. The mechanisms for adsorption were speculated to be physisorption and chemisorption for the removal of carbofuran by the biochar. The quadrupole interactions, dipole/induced dipole, van der Waals dispersion forces, π–π electron donor–acceptor interactions, and hydrogen bonding via H donor–acceptor interactions were the underlying mechanism(s) behind the sorption of carbofuran by biochar.

Fernandes et al. (2021) used a sugarcane-filtered cake to generate biochar for the adsorption of thiamethoxam pesticide from wastewater. The biochar was prepared by slow pyrolysis at 380°C for 2 h in a bench reactor containing nitrogen gas. Approximately 136 g of biochar was obtained after pyrolysis, and the mass yield derived was 68%. SEM analysis identified blocked holes that were generated due to the ash content and the temperature used in the biochar preparation. The surface area of the biochar was approximately 19.8 m^2^/g, and the pore volume and average pore diameter were 0.087 cm^3^/g and 172 Å, respectively. The functional group analysis via FTIR revealed O–H stretching (3,696.82, 3,621.11, and 3,413.47 cm^−1^), C=C stretching of the aromatic ring (1,615.09 and 1,429.85 cm^−1^), phosphites P=O and P–O–C and C–O–C stretching (1,094.74, 1,035.54, 1,009.78, and 914.01 cm^−1^, respectively), and C–H aromatic bending (773.03 cm^−1^). These functional groups were responsible for the π–π interactions of heteroaromatic rings, that is, thiamethoxam and aromatic rings from biochar, and also, hydrogen bond interactions between the biochar hydroxyl group and thiamethoxam molecules, followed by different dipole interactions. The monolayer and homogeneous adsorption of thiamethoxam on biochar was revealed from the Langmuir isotherm model, with *R* ^2^ = 0.9975 and a maximum adsorption of approximately 10.17 mg/g. Approximately 70% of adsorption was accomplished within 60 min of reaction time, with the concentration of the adsorbent being 10 mg/L. The adsorption process followed a pseudo-second-order model.

Wang et al. (2020) synthesized biochar from tangerine seed-activated carbon (TSAC) for the removal of carbamate pesticides (CMs). The biochar was pyrolyzed for 1–6 h in a muffle furnace at temperatures 500°C–900°C. The specific surface area of the biochar was 659.62 m^2^/g, and the pore volume and pore diameter of the biochar were 0.6203 cc/g and 1.410 nm, respectively. SEM revealed pores and rough structures. An increase in carbonization temperature resulted in a decrease in the yield of biochar. The highest yield of 42.61% was observed for TSAC-1 (700°C) within 1 h. TSAC-8 (600°C, 4 h) had the highest removal efficiency of 99.49% for carbaryl. FTIR revealed peaks specific to O–H and C–H (3,300 and 2,900 cm^−1^), C=O (1746 cm^−1^), and C–O (1,060 and 1,160 cm^−1^). The highest removal of CMs was observed at pH 7, 20°C, an adsorbent dose of 2 g/L, a pesticide concentration of 10 mg/L, and a contact time of 15 min. The adsorption was spontaneous and exothermic with a low degree of randomness. The adsorption kinetics followed the pseudo-second-order model (*R* ^2^ > 0.993). The adsorption isotherm followed the Langmuir model (*R* ^2^ = 0.9826–0.9999), indicating monolayer sorption. The adsorption capacities of TSAC for CMs by Langmuir were in the order methiocarb (93.46 mg/g) > carbaryl (44.64 mg/g) > pirmicarb (39.37 mg/g) > isoprocarb (13.95 mg/g) > metolcarb (9.11 mg/g) > bendiocarb (7.97 mg/g). Even after three regeneration cycles, TSAC showed the highest adsorption capacity for carbaryl, methiocarb, and pirimicarb, indicating its reusable nature.

### 2.4 Pharmaceutical products

The indiscriminate use of antibiotics and their release in natural resources can lead to severe health threats due to their potential role in multidrug resistance in various pathogens. Pharmaceutical products pollute the environment after being released in the effluents from hospitals, industries, and wastewater-treatment plants (Wang and Wang, 2016). Pharmaceutical products (such as analgesics, antibiotics, anticonvulsants, hormones, fragrances, *β*-blockers, antidepressants, and antiepileptics) can pose potential threats to the environment, flora, and fauna after entering the food chain (Cincinelli et al. 2015). Such pollutants can severely impair metabolism in both animals and plants, and can be a potential threat to human health. Microbial hazards include the significant risk of helminthiasis like ascariasis and giardiasis, enteropathogenic infections like cholera, typhoid, shigellosis, *H. pylori* and *E. coli* infections, listeriosis, salmonellosis, and enterovirus infections like rotavirus and poliovirus, which have been shown to directly correlate with inadequate treatment of wastewater polluted by pharmaceutical wastes. The chemical hazards to human and other animals include toxicological implications involving acute and chronic toxicity, carcinogenicity, and reproductive, developmental, and neuro toxicity (Koopaei and Abdollahi, 2017). Hence, several attempts have been made to remove these pharmaceutical products from effluents using biochars given in Table 4. The table summarizes the use of various agrowastes in developing biochars exclusively used for the removal of pharmaceutical products. Additionally, various parameters, such as pH, temperature, contact time, or duration, important for determining the efficiency of the biochar in removing the pharmaceutical products, are also summarized in the following.

**TABLE 4:** Agrowaste-generated biochar for the removal of pharmaceutical products.

Biochar sources	Pharmaceutical products	pH	Temperature (°C)	Time	Removal efficiency	Reference
Bagasse, bamboo, and hickory chips	Sulfamethoxazole (SMX) and sulfapyridine (SPY)	6	-	8–12 h	83.3% (SMX) and 89.6% (SPY)	Huang et al. (2020)
Food scrap	Tetracycline	7	-	-	8.23 mg/g	Hoslett et al. (2021)
Cotton gin waste (*Gossypium L.*) and guayule bagasse (*Parthenium argentatum*)	SPY, docusate (DCT), and erythromycin (ETM)	7 and 10	-	After 24 h	70% (SPY), 98% (DCT), and 74% (ETM)	Ndoun et al. (2021)
Rice hull	Sodium diclofenac	2	-	35 min	96%	Filipinas et al. (2021)
Rice husk	Tetracycline	9	25	192	552.0 mg/g	Chen et al. (2018)
Wheat straw	Norfloxacin (NOR)	5–11	-	-	25.53 mg g^-1^	Zhang et al. (2018a)

Huang et al. 2020 developed biochar from three different raw materials, bagasse (BG), bamboo (BB), and hickory chips (HC), for the removal of two sulfonamide antibiotics, sulfamethoxazole (SMX) and sulfapyridine (SPY). The biochars were prepared by pyrolyzing the biomass at three different temperatures, 300°C, 450°C, and 600°C, in a N_2_-filled tubular furnace for 1.5 h, and the obtained products were named BB300, BB450, BB600, BG300, BG450, BG600, HC300, HC450, and HC600, respectively. The ball-milled biochar was obtained in an agate jar after grinding with Gballs, and the resulting biochars were denoted as BM-BG300, BM-BG450, and so on. FTIR analysis revealed that the ball milling increased the surface functional groups, such as –CH_2_ (2,920 cm^−1^), C=C (1,597 cm^−1^), C–O (1,696 cm^−1^), –CO (1,262 cm^−1^), and C–H (882, 820 and 762 cm^−1^). A higher adsorption efficiency was observed for the biochars obtained by ball milling at 450°C. The ball milling increased the removal efficiency from 33.4% to 83.3% of SMX for BM-HC450 and from 39.8% to 89.6% of SPY for BM-BB450 due to an increase in surface functional groups or surface-specific area. The highest adsorption ability for SPY was observed for BM-BB450, and for SMX, it was observed for BM-HC450. Although these biochars are efficient in the removal of the pollutants, their scalability needs to be ensured. Moreover, minimum air pollution while developing the biochar should be kept in mind while scaling up to meet its applicability in real-world scenarios. An increase in the pH resulted in decreased adsorption. Hence, pH 6 was optimum. SMX and SPY removal by BM-HC450 and BM-BB450, respectively, was achieved at a contact time of 8–12 h. The SMX adsorption by BM-HC450 was approximately 75% within 8 h, while SPY adsorption by BM-BB450 was 80%. A kinetics study found that the Elovich model (*R* ^2^ greater than 0.96) was well fitted for the adsorption, which indicated that the adsorption was a multiple mechanism process. The adsorption isotherms were determined for these two biochars by adding 5–70 mg BM-HC450 and BM-BB450 into 10 mg/L SPX or SPY solutions. The Langmuir model (*R* ^2^ = 0.98 for SMX and 0.96 for SPY) was a better fit than the Freundlich model, and the maximum adsorption efficiency observed by the Langmuir model was 100.3 mg/g (SMX) and 57.9 mg/g (SPY). In the adsorption isotherm studies of wastewater (pH 7.6), the Freundlich model was best fit for SMX (*R* ^2^ = 0.98) and SPY (*R* ^2^ = 0.95). The Langmuir maximum adsorption capacity obtained for SMX and SPY was 25.7 mg/g and 58.6 mg/g, respectively. The π–π interaction, hydrophobic interaction, and electrostatic interaction might be the underlying mechanism behind the sorption of SMX and SPY by BM-HC450 and BM-BB450. Such interactions can be exploited for real-time application in biochar-mediated removal of pharmaceutical wastes. This strategy can be implemented in industries, hospitals, and research institutes as well, where the effluents have a higher load of antibiotics.

Hoslett et al. (2021) produced agriculture waste- and food scrap-derived biochar (including plant trimmings and other materials such as vegetables and fruit peels) for the removal of tetracycline from water. The biochar was prepared by pyrolyzing biomass at 300°C for 12 h. EDX analysis identified a high amount of potassium present in the biochar. SEM images showed the various sizes of the pores on the biochar surface. FTIR analysis showed peaks associated with C–H (alkane) and –OH (alcohol) (3,000 and 2,800 cm^−1^, respectively), C=O and C=C (1750 and 1,500 cm^−1^, respectively), C–H (methylene group) (1,465 cm^−1^), and C–O (primary alcohol) and C=C (alkene) (1,100 and 900 cm^−1^) functional groups. Raman spectroscopy indicated a mixture between carbon structures, where the peak at 1,370 cm^−1^ was attributed to the disordered graphitic structure (D), and the peak at 1,590 cm^−1^ represented an ordered graphitic structure (G), including sp^2^ and sp^3^ hybridization. An adsorption capacity of biochar between 2.98 and 8.23 mg/g was obtained with initial tetracycline concentrations of 20 and 100 mg/L, respectively, at a neutral pH of 7. The adsorption isotherm showed that the Freundlich isotherm model was the best fit for the adsorption of tetracycline, which indicated the dominance of the multilayer adsorption process. A kinetics study revealed that the adsorption isotherm followed the Elovich model, where the intra-particle diffusion and liquid film diffusion controlled the rate of transfer of tetracycline into the adsorbent. The π–π EDA interaction due to the aromatic ring and C=C bond was the underlying mechanism behind the adsorption of tetracycline, which might be similar to the previously observed adsorption for SMX and SPY.

Ndoun et al. (2021) produced biochar from cotton gin waste (*Gossypium L.,* denoted as CG) and guayule bagasse (*Parthenium argentatum,* denoted as GB) for the removal of sulfapyridine (SPY), docusate (DCT), and erythromycin (ETM) from an aqueous solution. The CG and GB biochar was prepared by pyrolizing 0.5–1.5 kg of biomass using a gas-tight retort at a low heating rate (0.05°C–0.1°C), with three different temperatures of 350, 500, and 700°C for 2 h in the presence of N_2_ gas. The obtained biochar was named GB350, GB500, GB700, CG350, CG500, and CG700. The negative zeta potential of all the biochars was increased by increasing the pH. BET analysis confirmed a low surface area and a pore volume of GB700 (at 5.92 m^2^ g^−1^), GB500 (at 0.06 m^2^ g^−1^), and GB350 (at 0.00 m^2^ g^−1^) compared to CG700 (at 16.33 m^2^ g^−1^), CG500 (at 2.06 m^2^ g^−1^), and CG350 (at 2.40 m^2^ g^−1^). The surface area could be increased by increasing the pyrolysis temperature. The functional groups present in the biochar were –OH (3,400 and 3,500 cm^−1^), C=O (1770 cm^−1^), C=C (1,400 and 1,500 cm^−1^), C–O (1,350 cm^−1^), and C–H (2,850–2,960 cm^−1^). The peak at 870 cm^−1^ was attributed to the C–H bending vibration of the β-glycosidic linkage. The maximum SPY removal (70%) by CG700 at pH 8.3–11.2 after a contact time of 24 h was due to hydrophobic interactions. Another mechanism responsible for SPY removal was the formation of negative charge-assisted H-bonds between the anionic SPY and the O-containing functional groups. The π–π EDA interaction due to the presence of amino functional groups, N or O-hetero-aromatic rings, and C=C, OH, and C=O, which act as an electron donor, was another predominant mechanism. More O-containing functional groups, lower surface area, and pore volume indicated GB biochars’ low SPY removal efficiency due to higher hydrophilicity, which limits the potential for hydrophobic interactions. The highest removal efficiency of DCT of 98% by CG700 compared to GB was due to hydrophobic interactions. The maximum DCT removal was obtained by BG350 compared to BG700 due to the higher content of natural organic matter (NOM), which may play an important role in the interaction between DCT and BG. The ETM adsorption was high for CG700 (74%) compared to GB700 (53%) at pH 10.2–11.4. The main mechanism behind the adsorption of ETM was the hydrophobic interaction formed by van der Waals forces between the hydrophobic ETM and biochar. The ETM adsorption was higher in GB350 (50%) and GB500 (64%) than in CG350 and CG500 due to O-containing groups, which increased the formation of H-bonds. A pseudo-second-order model (*R* ^2^ ˃ 0.8) was well fitted for the SPY, DCT, and ETM adsorption. The adsorption isotherm revealed that the Langmuir model was a better fit than the Freundlich model. The maximum adsorption of SPY, DCT, and ETM was obtained at pH 7 and 10 after 24 h.

Filipinas et al. (2021) developed a rice hull-derived biochar (RHB) for the removal of sodium diclofenac (SD) from an aqueous solution. RHB was prepared by drying the rice hull at 100°C for 30 min and pyrolyzing at a high temperature, followed by sieving to obtain 300–500-µm particles, which were then torrefied in an oven at 350°C for 1 h. EDX analysis confirmed that the raw rice hulls were mainly composed of C (16.53 wt%), O (40.51 wt%), and Si (42.96 wt%), in which C was significantly increased after torrefaction (RHB) from 25.3% to 40.48%. The absorbance peak in the FTIR spectra at 3,300–3,400 cm^−1^ represented the –OH group due to SiOH and adsorbed water, while those at 3,000 cm^−1^ and 2,100–2,400 cm^−1^ represented C–H and C≡C stretching. The peak at 700 cm^−1^ was for the Si–O bond, and the peak at 1,700 cm^-1^ represented C=O, which shifted to 1,900 cm^−1^ after SD adsorption, indicating the role of the C=O bond in the carbonyl or carboxyl groups in SD adsorption. The highest SD removal of 96% (3.3 mg g^−1^) was achieved at pH = 2, an initial SD concentration of 100 mg L^−1^, an RHB dosage of 0.5 g, and a contact time of 35 min. The adsorption isotherm determined that the Langmuir isotherm model was better fit at pH 2 (*R* ^2^ = 0.9827) and pH 7 (*R* ^2^ = 0.9460) for Sd adsorption onto the RHB surface. A kinetic study revealed that the pseudo-second-order model (*R* ^2^ = 0.9999) was well fit.

Chen et al. (2018) developed H_3_PO_4_-modified rice straw biochar for the removal of tetracycline (TC) from an aqueous solution. The biochar was prepared by pyrolyzing the biomass in a muffle furnace at 700 °C for 2 h and was further treated with a H_3_PO_4_ solution for modification. The raw biochar was labeled as rice straw biochar (RC), and the modified biochar was named RCA. BET analysis showed a minor increase in the surface area, from 369.26 m^2^/g in RC to 372.21 m^2^/g in RCA, after modification by H_3_PO_4_. The pore volume for RC and RCA remained the same (V_t_: 0.23 cm^3^/g, V_mic_: 0.09 cm^3^/g, and V_mes_: 0.14 cm^3^/g). The ratio of O/C, (O+ N)/C, and H/C of RCA decreased compared to RC, which indicated that after H_3_PO_4_ modification, the biochar became less hydrophilic with weaker polar groups. FTIR analysis revealed the absorbance peaks of –OH (3,436 cm^−1^), C=C and C=O (1,591 cm^−1^), C–O (1,400 cm^−1^), and SiO_3_^2−^ (1,050 cm^−1^), while no change in the functional group after H_3_PO_4_ modification was found. EDS analysis identified that Ca, Mg, F, and Na were lost after H_3_PO_4_ modification. The effect of pH on the adsorption of TC by RCA was tested at pH ranging from 5 to 9, and the highest adsorption was observed at pH 9, which was approximately 552.0 mg/g. The equilibrium time for both the biochars was 192 h. A pseudo-second-order model (*R* ^2^ = 0.901–0.911) was a better fit than the pseudo-first-order model. The adsorption isotherm by Langmuir and Freundlich isotherm models had a similar *R* ^2^ value of 0.981–0.986 (Freundlich) and 0.973–0.981(Langmuir). The q_max_ of TC according to the Langmuir model for RCA was 167.5 mg/g. It was found that after H_3_PO_4_ modification, the adsorption efficiency of RCA increased compared to RC. The underlying mechanism behind the adsorption of TC on the surface of biochar might have included cation exchange, π–π EDA interactions, H bonding, electrostatic interactions, and surface complexation.

Zhang et al. (2018b) developed montmorillonite–biochar (MT-BC) composites from wheat straw for the removal of norfloxacin (NOR) and to check the effect of humic acid (DHA) and Cu^2+^ on NOR adsorption. The MT-BC composite was prepared by using a mixture of montmorillonite and wheat straw in a muffle furnace at 400°C in the presence of N_2_ flow for 6 h, and the same process was followed to develop wheat straw biochar (BC) without montmorillonite. SEM images showed the porous surface morphology of MT-BC. EDX analysis confirmed higher C (58.8%) and O (27.0%) content in MT-BC compared to BC. The lower atomic ratio of H/C indicated the higher aromatic structure of MT-BC. The polarity index (N + O)/C of MT-BC was 0.37. BET analysis revealed a higher surface area (112.6 m^2^ g^−1^) and pore volume (0.604 cm^3^ g^−1^) of MT-BC compared to BC. The FTIR spectra exhibited peaks at 1,640 cm^−1^ associated with the C–C band, 660 cm^−1^ specific to mineral compounds such as carbonates and phosphates, and 1,190 cm^−1^ for the C–H and OH deformation of the COOH or C–O bond. These observations indicated increased O-containing functional groups on biochar after montmorillonite modification. The maximum adsorption ability was observed in MT-BC, which increased from 10.58 mg g^-1^ to 25.53 mg g^−1^, compared to BC, at an optimal pH of 5–11. The adsorption of NOR was suppressed in the presence of DHA and Cu^2+^ (20–150 mg L^−1^), which indicated the role of DHA and Cu^2+^. The O-containing group of biochar might have formed a H-bond with NOR moieties, and pore-filling was the major factor for the enhanced adsorption affinity for NOR. Thus, the H-bonds, electrostatic interactions, and pore-filling were the underlying mechanisms behind NOR adsorption by MB-BC. The Langmuir model was a better fit than the Freundlich model, which indicated that the adsorption was a monolayer process.

### 2.5 Polycyclic aromatic hydrocarbons

Polycyclic aromatic hydrocarbons (PAHs) are highly toxic mutagens and potential carcinogens. These organic pollutants are ubiquitously found in the environment as a result of the incomplete pyrolysis of organic matter during various anthropogenic processes, such as the combustion of fossil fuels and organic wastes (Hu et al., 2014). Biochar-mediated removal of PAHs seems to be a promising solution, which is given in Table 5.

**TABLE 5:** Agrowaste-generated biochar for the removal of PAHs.

Biochar source	Hydrocarbon	pH	Temperature	Time	Removal efficiency	Reference
Coconut waste and orange waste	PAHs	-	-	180 min	51.26%–70.66%	De Jesus et al. (2017)
Peanut shell	PAHs	-	-	30 min	-	Yin et al. (2019)
Rice husk	Chlorobenzene (CB)	3	25°C	6 h	-	Zhang et al. (2018a)
Wheat straw	PAHs	-	-	-	70%	Kong et al. (2021)
Wheat straw	Petroleum hydrocarbons	-	-	-	-	Li et al. (2019b)

De Jesus et al. (2017) evaluated the PAH removal efficiency of different agricultural waste-based biochars, like coconut waste biochar (BCW) and orange waste biochar (BOW). The biochars were prepared by pyrolyzing the dried biomass in a furnace at 350°C. The infrared spectrum of CW revealed the bands of OH (3,409 cm^−1^), C–H stretching (2,921 cm^−1^), CO_2_ present in the air (2,369 and 2,348 cm^−1^), C=O stretching (1732 cm^−1^), C=C aromatic stretching (1,508 cm^−1^), C–H aromatic vibrations (786 cm^−1^), and C–O ether stretching (1,060 cm^−1^). Similarly, for the OW, the bands of OH (3,407 cm^−1^), aliphatic CH stretching (2,926 cm^−1^), CO_2_ (2,362 and 2,343 cm^−1^), C=O bond stretching (1,742 cm^−1^), C=C stretching (1,520 cm^−1^), C–H vibration (762 cm^−1^), and C–O ether stretching (1,058 cm^−1^) were identified from the FTIR spectrum. After the formation of BCW and BOW, the reduction in the hydroxyl band intensity at 3,409 cm^−1^ and the disappearance of the C–O stretching ether band at 1,060 cm^−1^ were noted. Elemental analysis identified the lower ratios of H/C in BCW and BOW, which might cause higher PAH adsorption capacities. The surface area and mean porosity were 233.869 m^2^/g and 2,186.5 Å for BCW and 261.233 m^2^/g and 4,299 Å for BOW, respectively. The PAH adsorption efficiencies of BCW and BOW were 41.26%–86.09% and 22.73%–88.19%, respectively, while the mixed-solution adsorption efficiencies were 51.96%–83.92% (BCW) and 23.84%–84.02% (BOW), respectively. An equilibrium time of 180 min was noted for adsorption from the mixed solution. The mechanism behind the adsorption of PAHs on biochars was the interaction between adsorbents and π–π electrons of aromatic compounds and, also, the direct physical adsorption of compounds on the BC surface. Isothermal studies of the adsorption showed that the Freundlich model with *R* ^2^ = 0.7232–0.9852 was a better fit than the Langmuir model and, thus, confirmed the heterogeneous surface adsorption. The adsorption process followed a pseudo-second-order model. Desorption efficiencies ranging from 40.62% to 52.18% for BCW and 51.26%–70.66% for BOW were noted. The same BCs showed potent adsorption ability even after two adsorption–desorption cycles.

Yin et al. (2019) developed a new method for the removal of PAHs from water using peanut shell biochar (PSBC) fabricated using 2 g of PS powder carbonized at 800°C for 2 h in a N_2_-containing furnace. The powder obtained was further rinsed and dried at 80°C for 12 h. SPME fibers were developed by sonicating stainless-steel wires (SSWs) in methanol for 15 min, followed by sonication in acetone for another 15 min and a further 10 min in methanol. The SSW was dried at room temperature for 1 h, and 1 cm of the wire tip was submerged in a mixture of 0.25 g neutral multipurpose sealant and 1.5 mL chromatography-grade hexane. The mixture on the tip was solidified by heating the wire at 100°C for 30 min. The coated wire was aged in nitrogen ambience at 250°C for 1 h. SEM analysis revealed the porous structure of the fiber, with a diameter and thickness of 182 µm and 27.5 µm, respectively. The crystalline nature was identified from XRD analysis. The elemental studies via XPS and ES detected the presence of 56.15% carbon content and a low H/C ratio (0.03) for the PSBC. The parameters for the SPME process, such as the extraction time, were optimized at 30 min, while the optimized extraction temperature was in the range of 30°C–70°C. The optimal desorption temperature was 260°C, while the salt concentration was 0.25 mg/L NaCl. The recoveries of PAHs from pond water using SPME fibers ranged between 82.34% and 116.20%, while from river water, it was between 81.02% and 110.84%.

Zhang et al. (2018b) derived biochar from rice husk and studied the potential degradation of chlorobenzene (CB). Initially, the rice husk was washed and dried at 105°C overnight. The biochar was then pyrolyzed at 550°C in a laboratory pipe oven under N_2_ flow at 2 mL/min for 2 h. The biochar obtained after cooling to room temperature was denoted as R550. Another type of biochar is oxi-R550, developed by oxidizing the biochar using H_2_O_2_. The oxi-R550 was fabricated by the treatment of 50 g/L biochar with 5% H_2_O_2_ for 24 h, followed by heating the suspension for 5 h at 80°C to remove H_2_O_2_. The product obtained was dried at 80°C for dehumidification. Batch experiments were carried out for the degradation of CB using R550. Initially, 0.1 g biochar and 10 mL CB (20 mM) were mixed at different pHs and at 25°C temperature. SEM analysis showed the porous structure of R550 containing mineral-coated inner surfaces of macropores and micropores, while the outer surface was dotted. XRD analysis showed the amorphous structure of the biochar. The functional groups present on the surface of biochar were –OH (3,429 cm^−1^), –CH_2_ (2,932 cm^−1^), O–H with an aromatic ring (1,378 cm^−1^), C–O–C (1,205 cm^−1^), COOH/COOC (1,700 cm^−1^), C=O (1,586 cm^−1^), and Si–O–Si (1,087, 800, and 460 cm^−1^). The redox ability of the biochar was developed due to the presence of the hydroquinone–quinone moiety on the surface of the biochar. The CB degradation efficiency of R550 initially showed a rapid increase in the first 6 h of contact and then gradually decreased at a prolonged time of 10 h. At 6 h of contact time, the removal efficiency of CB was 59.4% (pH 3), 42.2% (pH 7), and 25.9% (pH 11). A decrease in the phenolic–OH peak with a concomitant increase in C=O quinone peak was noted, which is supported by the proposed degradation pathway. R550 followed second-order kinetics during CB degradation. The possible mechanism behind CB degradation using R550 was the formation of ^•^OH radicals.

Kong et al. (2021) investigated the synthesis and use of biochar (BC) derived from wheat straw (WS) for the removal of PAHs from contaminated soils. The biochars were prepared by pyrolyzing the raw material at 500°C for 3 h. The biochar obtained was labeled as WS500 on the basis of the temperature used for the pyrolysis. The heating rate during the pyrolysis was approximately 10°C/min. Three different soil treatments were performed, where the first sample CK contained soil contaminated with Luquan (LQ), Jingxing (JX), and Jinzhou (JZ). The second sample BC + NaN_3_ (contaminated soils with 5% (w/w) BC and NaN_3_ solution) and the third sample BC (contaminated soils with 5% (w/w) biochar) were also used. The samples were incubated for 84 days, followed by freezing and evaporation to detect the total petroleum hydrocarbons (TPHs) and 16 prior PAHs, respectively. A low yield of biochar of approximately 25.17% was obtained. A high carbon content of approximately 73.95% (w/w) was obtained from elemental analysis. The specific surface area and pore volume of the BC were 2.37 m^2^/g and 0.005 cm^3^/g, respectively. FTIR analysis revealed different functional groups present on the biochar surface, such as –OH groups of water or phenolic C–OH group stretching (3,448 cm^−1^), carboxylic acid C=O stretching (1,578 cm^−1^), and the C/O out of plane deformation from carbonates and alkyl bends (618 cm^−1^). SEM analysis confirmed the honeycomb structure of the biochar. An approximately 70% decrease in PAH content of soil was noted after adding CK. The soils containing both BC + NaNO_3_ increased PAH reduction by 10.25%. Microbial community composition analysis revealed that the majority of the sequences analyzed were from the phylum Proteobacteria. The addition of WS500 to the soil increased PAH degraders, which aided in PAH biodegradation.

Li et al. (2019a) analyzed the long-term effect of biochar derived from wheat straw on the removal of petroleum hydrocarbons from the soil. The raw material of the biochar was pyrolyzed at 600°C in a muffle furnace to obtain the wheat straw biochar (SB). The soil microbial fuel cell (MFC) was filled with the SB at a 2% mass ratio. The control sample was denoted as CK (without biochar). The soil MFC contained a homogeneous mixture of dried soil and 100 g biochar in 40 mL of distilled water. There was no addition of exogenous inoculation or buffer solution to the MFC. The total petroleum hydrocarbon (TPH) removal efficiency of SB was 15%–17% higher than that of CK. The aromatic, polar materials and asphaltene were easily degraded by SB, which was about 26%–36% higher than that of CK. The degradation of all 16 PAH increased by 38% compared to the CK. In the analysis of bioelectricity generation via soil MFC, the highest voltage of 173.9 ± 2.3 mV was noted for SB on day 191 of incubation. The rate of bioelectricity generation by SB was approximately 0.021 C/g/day. The capacitance of SB MFC (1.5 × 10^−2^ F) was 46% higher than that of CK (1.0 × 10^−2^ F). An increase in *Actinotalea* by 144%–263% was noted due to the application of SB in soil. Furthermore, the methanogenic degradation of PAHs was reported by different species, such as *Methanosarcina*, *Methanoculleus*, *Halovivax*, and *Natronorubrum*.

## 3 Conclusion

This article discusses agrowaste-derived biochar, along with its nanotextured surface features and applications. Although various methods are employed during the production of the biochar, slow pyrolysis is established to be the most promising method, providing maximum yield. The physical and chemical properties of the biochar are exploited for effective environmental bioremediation. Large surface area (∼500 m^2^/g), more porosity (∼4,000 Å), and high adsorption capacities (180 mg/g) have enabled the biochar to remove hazardous dyes, such as Patent Blue (V), Reactive Black 5, and Congo red. In particular, positively charged dyes, like brilliant green, can be removed more effectively (∼99%) by employing biochar generated from agrowastes of oil-producing plants, such as soybean straw. Similarly, biochar generated from the peels of avocado, banana, and other plant wastes can effectively remove hazardous metals, such as lead, chromium, cadmium, and arsenic. The biochars can also remove pesticides, such as chlorpyrifos, diazinon, atrazine, and imidacloprid, which are mostly recalcitrant in nature. Organophosphorus pesticides are more selectively removed using biochar with an efficiency equivalent to∼98%. Furthermore, pharmaceutical waste, like docusate, erythromycin, sulfamethoxazole, sulfapyridine, and tetracycline, can be effectively adsorbed on the biochar and removed from effluents. Last but not least, most significantly, PAHs can be remediated by agrowaste-generated biochar. However, PAH removal by nanocomposites prepared using iron oxide and chitosan is more efficient, with efficacies ranging from 92% to 95%, against anthracene and phenanthrene, which are significantly higher than that of biochar (∼70%). Hence, thorough optimization of the process parameters, such as dosage of the biochar, initial concentration of the pollutants, contact time, pH, and appropriate surface treatment, along with an understanding of the kinetics and underlying mechanism, would certainly help develop a community-based wastewater-treatment process. This will ensure a clean environment by effectively removing hazardous pollutants from the environment and can minimize their adverse effects on the biodiversity and human health.

Due to the significant generation of agrowaste globally, the biochar from such materials can be considered an important strategy for circular economy. Hence, more studies should be aimed at scaling up agrowaste-mediated biochar production employing three reactors (semi-pilot, pilot, and industrial). Biochar generation conditions in the semi-pilot reactor can ascertain the optimal production temperature favoring a high carbon content, moderate pH, and maximum pollutant removal efficiency. Industrial-scale study will facilitate improved carbon stability that is critical for the biochar to serve as a carbon sink. The two most important factors needing control during scale-up studies are temperature and retention time.

## 4 Future prospects

**FIGURE 3:**
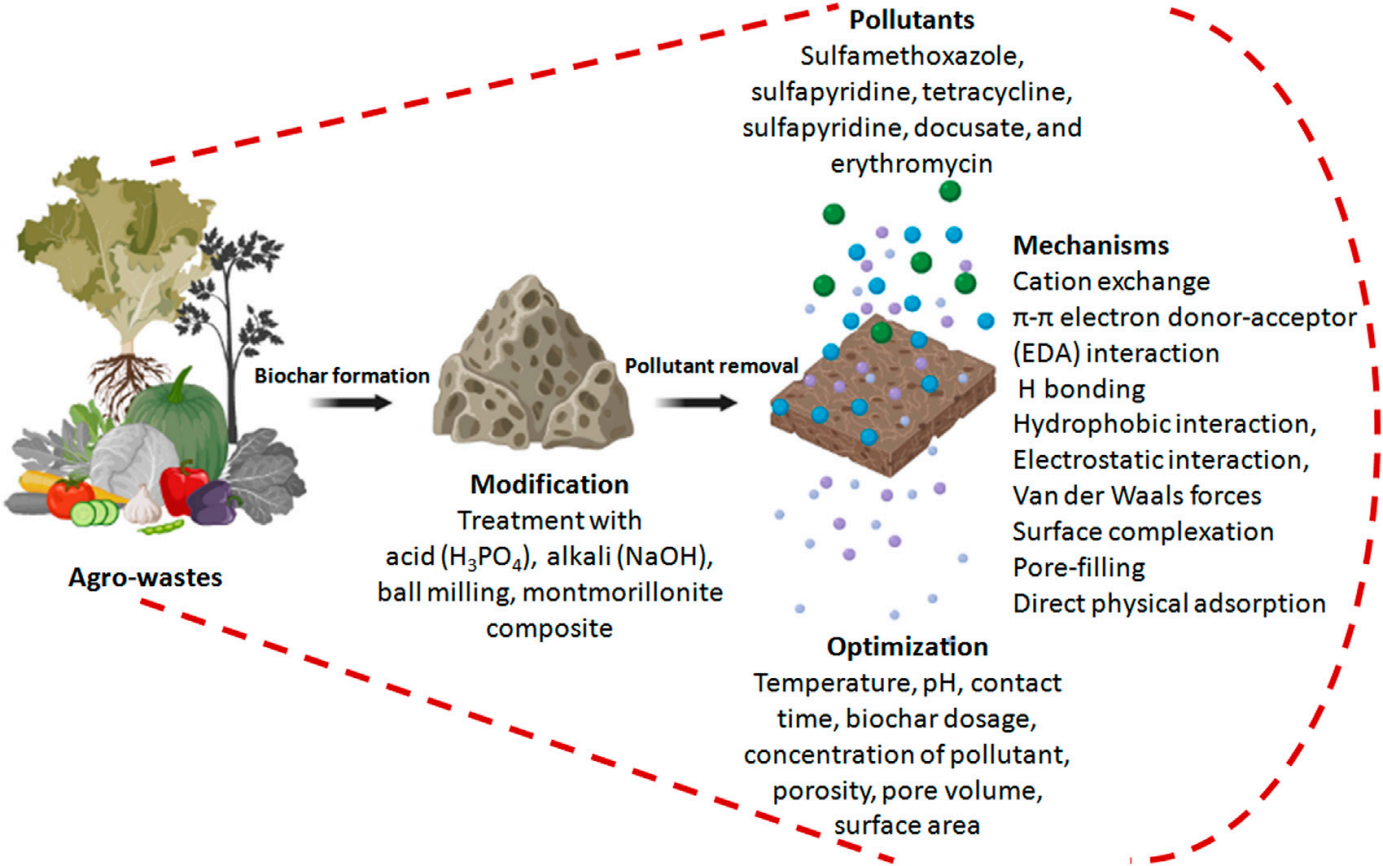
Agrowaste-mediated biochar generation and future prospects toward surface modification and optimization for enhancement of pollutant-removal efficiency.

Agrowaste-mediated generation of biochar with nanotextured surfaces is an economically viable and sustainable green approach that can have multidimensional industrial applications, as shown in Figure 3. The unique surface-associated functional groups and large surface area are most ideal for facilitating catalytic reactions. The nanotextured surfaces of the biochar can be activated using appropriate treatments, functionalized using specific ligands, and/or incorporated within nanoclay for further synergistic enhancement of their catalytic properties (Esteves et al., 2020; Munir et al. 2020). Unlike single- and multi-walled carbon nanotubes, graphene oxide, or activated carbon, which are often used as supercapacitors and/or in energy storage, biochar can be used as a cheaper and effective electrode material (Gupta and Khatri, 2019). Its inexpensive nature, porous structure, and attractive surface properties can be exploited for developing microbial fuel cells (Thomas et al., 2019). The two forms of carbon in biochars (liable and recalcitrant) can be microbially mineralized. Hence, there is a need to explore biochar-associated carbon sequestration in soil and the mitigation of greenhouse gases (Li and Tasnady, 2023; Shakoor et al. 2021; Brown et al., 2020). Biochar can also be included in animal feed as it enhances immunity, reduces the risk of botulism, improves digestion, enhances the growth rate, and decreases methane formation (Masrom et al., 2018). In view of this background, it is clear that agricultural waste can be converted to biochar to meet the requirements in various industrial sectors, which include the environment, agriculture, food, and pharmaceutics.
